# Chiropractors` experience and readiness to work in Indigenous Australian Communities: a preliminary cross-sectional survey to explore preparedness, perceived barriers and facilitators for chiropractors practising cross-culturally

**DOI:** 10.1186/s12998-017-0144-0

**Published:** 2017-05-02

**Authors:** Dein Vindigni, Barbara I. Polus, Sonja Cleary, Aunty Kerrie Doyle

**Affiliations:** 1 Discipline of Chiropractic, School of Health and Biomedical Sciences, RMIT University, Bundoora West Campus, Plenty Road Bundoora, Victoria, 3083 Australia; 2 Discipline of Nursing, School of Health and Biomedical Sciences, RMIT University, Bundoora West Campus, Plenty Road, Bundoora, 3083 Victoria Australia

**Keywords:** Chiropractors, Experience, Indigenous, Australian, Cultural competency

## Abstract

**Background:**

Indigenous people make up approximately 3% of the total Australian population and score poorer on all health indices, including back pain. Chiropractors are well placed to alleviate back pain, yet there is no research that considers chiropractors’ readiness to treat Indigenous patients. This study explores chiropractors` experience working with Indigenous Australians, describes perceived barriers and facilitators to chiropractors’ participation in Indigenous Healthcare and their willingness to engage in cultural competency training.

**Methods:**

This study used a non-representative cross-sectional design and a convenience sample. Participants were recruited via email invitation to complete an online survey and encouraged to send the invitation on to colleagues. A 17-item online-survey measured demographic data, perceived barriers and facilitators related to caring for Indigenous Australians, participants` level of comfort when working in Indigenous health, and their willingness to participate in cultural competency programs to enhance their skills, knowledge and cultural capacity when engaging with Indigenous Australians. Analysis of the data included descriptive statistics as well as thematic analysis of qualitative free text.

**Results:**

One hundred and twenty-five chiropractors participated in the survey. The majority of participants (86%, *n* = 108) were employed in private practice. 62% of respondents were members of the Chiropractors' Association of Australia, 41% were Chiropractic and Osteopathic College of Australasia members. 60% of chiropractors considered that they had, or do treat Indigenous patients yet only 4% of respondents asked their patients if they identified as Indigenous. A majority of participants expressed a high level of ‘comfort’ or confidence in working with Indigenous people while only 17% of respondents had undertaken some form of cultural proficiency training. A majority of respondents (62.7%, *n* = 74) expressed an interest in working with Indigenous Australians and a majority (91%, *n* = 104) were willing to participate in training to develop Indigenous cultural competency.

**Conclusions:**

The study points to a need for chiropractors to have access to cultural proficiency training in order to develop the capability and confidence to engage respectfully with their Indigenous patients. This preliminary study has provided the researchers with valuable insights aiding the development and implementation of an Indigenous cultural proficiency program for chiropractors.

**Electronic supplementary material:**

The online version of this article (doi:10.1186/s12998-017-0144-0) contains supplementary material, which is available to authorized users.

## Background

The gap in all health and quality of life outcomes for Indigenous Australians continues to be of great concern [1] and this gap also exists in musculoskeletal disorders. Chronic back pain, for example, is experienced by 12.7% of Australians, with low back pain (LBP) being the second highest burden of chronic disease [2]. While there are no census data measuring LBP across all geo-locations of Indigenous Communities, Lin et al. [3] recorded back pain as ‘profoundly disabling’ in some Indigenous Communities, and Vindigni et al. [4] demonstrated that in regional and remote Indigenous communities, LBP is the most prevalent musculoskeletal condition with financial disadvantage, limited geographical access to allied health professionals (such as chiropractors, osteopaths and physiotherapists) and sociocultural barriers identified as contributing to the burden of illness. Thus accessible and effective strategies for managing musculoskeletal pain experienced by Indigenous peoples are indicated.

The explanations given for the ongoing gap in health status between Indigenous and non-Indigenous people have been well described in the published literature (e.g. [5, 6]). The underlying causes relate to disadvantage across a spectrum of determinants including include social, political, educational, economic, geographical and cultural determinants of health [7].

Given the poorer overall health status of Indigenous people, it might be expected that the use of primary health services would be higher for Indigenous than non-Indigenous peoples, but this is not the case [8, 9]. Some of the barriers to accessing primary health care in Indigenous communities include a lack of appropriate health services, affordability and social/cultural acceptability of the service [10].

Considering the population distribution of Indigenous Australian people and the spread of chiropractic clinics in urban, rural and remote Australia, there is an expectation that a lack of geographically accessible health services should not be a barrier to chiropractic care.

There are approximately 5000 registered chiropractors in Australia [11]. Of the 3310 chiropractors that are members of the Chiropractors’ Association of Australia (CAA), 322 (9.7%) have registered practices with postcodes classified as remote or very remote and 21.7% have registered practices with postcodes classified as outer regional (CAA database of geographical location of CAA members) While these figures do not include all registered chiropractors, the CAA remains recognised as the peak body representing the majority of chiropractors in Australia. In Victoria 754 chiropractors are members of the CAA. Of these, 169 (22.4%) have practice postcodes classified as outer regional or remote (CAA database of geographical location of CAA members). When examining the geographical location of Indigenous Australians it was found that only 20% of Indigenous people live in remote or very remote areas, with 43% living in regional areas, and 35% of all Aboriginal and Torres Strait Islander people living in urban areas [12]. Thus the geographical location of chiropractic practice closely matches the location of the majority of Indigenous peoples, suggesting that location is not necessarily a barrier to accessing care.

Another important and possibly overlooked barrier accounting for the continued gap in health status is the reluctance of Indigenous people to access health care [13] often because Indigenous people do not feel valued [14] or heard [15] by clinicians particularly in mainstream healthcare services that may not resonate with Indigenous peoples' health paradigms.

In addition previous experiences of institutionalised racism have been reported by Indigenous Australians as a deterrent to seeking treatment, particularly for conditions that might be stigmatised, such as back pain [3]. To help overcome these perceived barriers it is critical that healthcare professionals including chiropractors are culturally competent so that when Indigenous Australians seek their service the experience is a positive one where they feel valued and heard. Cultural competence is the awareness, knowledge and sensitivity to other cultures combined with a proficiency to interact appropriately with people from other cultures in a way that is congruent with the behaviour and expectations that members of a distinctive culture recognise as appropriate themselves [16].

Importantly, as cultural competency training for health professionals has more generally been shown to improve patient outcomes [17] it is imperative that health professions are versed in culturally responsive practice [18, 19]. This research, therefore, explores the potential need for developing this type of training program.

Furthermore, beyond the perceived potential for chiropractors to provide effective management of musculoskeletal conditions for Indigenous Australians, there is a need to understand the extent to which Victorian chiropractors may already provide treatment for Indigenous people, the issues they may face and the opportunities for increasing the reach and impact of chiropractic care for Indigenous Australian people. Anecdotal evidence suggests that there is an under-representation of chiropractors caring for Indigenous Australians. Though the majority of chiropractors work in private practice, the extent to which they care for Indigenous people is unknown.

The main purpose of this study is to inform the development of cultural proficiency programs. It explores the extent to which chiropractors routinely collect data that identify Aboriginal and/or Torres Strait Islander patients, chiropractors’ clinical experience and participation in Indigenous healthcare, their preparedness and perceived barriers and facilitators to caring for Indigenous patients and their interest in participating in cultural competency programs to enable them to acquire the necessary knowledge, skills and attitudes to achieve best practice in promoting Indigenous health.

## Methods

The Human Research Ethics Committee of the Royal Melbourne Institute of Technology (RMIT) University (BSEHAPP 06–15) approved the project, including its design and recruitment methods. The aims were met using a non-representative, cross-sectional design.

While predominantly quantitative, this survey used a mixed method approach, by asking participants to consider the barriers and facilitators of Indigenous people accessing chiropractic care, and included an opportunity for participants to add comments. Quantitative data were subjected to descriptive analysis only, with qualitative data subjected to thematic analysis [20].

### Participants

Chiropractors who were members of the CAA (Victoria branch) were recruited by an email inviting them to participate in the online survey. In order to increase recruitment, participants were encouraged to contact their Chiropractic and Osteopathic College of Australasia (COCA) colleagues to participate in the online survey via a ‘snowballing’ technique [21]. The inclusion criterion was that participants identified as a chiropractor.

### E-Survey

To meet the aims of the research, a17-item online-questionnaire was created using the Qualtrics platform (the survey instrument is available as an additional file (Additional file 1). The survey was purposively piloted by a cohort of four academic clinicians. They were asked to review the content and clarity of the survey with minor typographical changes recommended. The questionnaire had quantitative and qualitative components.

Informed consent was captured in the first test item, with respondents exiting the survey at that point if they so preferred. Participants were asked to complete questions that recorded demographic data, including number of years in practice and the use of first consultation documentation that recorded if their patients identified as Aboriginal or Torres Strait Islander. Questions enquired about participants level of openness and confidence or ‘comfort’ when working in Indigenous health as well as their willingness to participate in a cultural competency program designed to enhance their knowledge, skill and attitudes to improve cultural capacity when engaging with Aboriginal people. Participants’ level of openness was measured by a Likert scale where ‘1’ was ‘no’ or ‘never’ and ‘10’ was ‘extremely likely’ or ‘always’. Chiropractors’ level of comfort when working with Indigenous patients was measured also using a Likert scale with 1 being not at all comfortable and 10, completely comfortable. Open-ended questions gave participants the opportunity to comment on the perceived barriers and facilitators to caring for Indigenous Australians.

### Procedure

The Qualtrics survey link was sent to the Chiropractors’ Association of Australia, Victoria (CAAV) together with a short email invitation from the C.I. and a participant information sheet explaining the study. The CAAV sent the email invitation, survey link and information sheet to all CAAV active members, who then chose to participate or not. Participants were also invited to forward the link to other chiropractors, using a snowballing method. The survey was ‘live’ for six months. Participation in the survey was limited to one attempt per participant only by the survey software (Qualtrics).

### Data analysis

The bulk of the questionnaire was analysed using descriptive statistics to answer the research questions. The open ended qualitative responses from participants focussed on participants’ perception of barriers and facilitators to Indigenous people accessing chiropractic services, and their willingness to undertake cultural competency training for health professionals. This approach was informed by a previous study conducted in a large, rural Indigenous Community exploring the prevalence of musculoskeletal conditions which also identified a range of barriers and facilitators for Community members accessing allied healthcare. The barriers and facilitators included geographical accessibility, financial and socio-cultural considerations [4]. Though the current study primarily explored the delivery of health services from the perspective of chiropractors, these perceived barriers and facilitators commonly emerge in other studies evaluating delivery of healthcare interventions to Indigenous people [8] and helped to inform the development of questions as well as the thematic analysis from data extracted from respondents to our survey.

The responses of the current study were subjected to thematic analysis [20]. Using thematic analysis responses to each qualitative question were examined to find commonalities, and meaningful patterns, and placed into codes [20, 22]. These codes were then combined into themes to answer the research questions [23] around the barriers and facilitators to Indigenous people accessing chiropractic care and to consider chiropractors’ interest in developing additional knowledge, skills and behaviours in working with Indigenous peoples. One of us (AKD) who is an experienced qualitative researcher with significant experience in this field undertook the original analysis. A review of the analysis was conducted by DV. The review included examining responses for patterns and commonalities as well as the combination of coding into themes.

## Results

### Demographics

One hundred and twenty five participants completed the online survey (M =66, F = 59), with an average age of 42.7 years, (95% CI [31, 55]). The majority of participants (86%, *n* = 108) were employed in private practice, and the remaining 14% (*n* = 17) were employed elsewhere. Only 3.2% (*n* = 4) had below a Bachelor degree (see Table 1).

**Table 1:** Participants’ level of qualification

Qualification	Number	Percent
Diploma	3	2.4
Advanced Diploma	1	.8
Bachelor (or Double Bachelor)	59	47.2
Masters	52	41.6
PhD	8	6.4
Did not respond	2	1.6

(*N* = 125)

Only 5 participants did not belong to a chiropractic organisation (see Table 2).

**Table 2:** Membership of a chiropractic organisation

Organisation	Number	Percent
COCA	60	48
CAA	77	61.6
Other^a^	18	14.4
None	5	4

(*N* = 125)

*COCA* Chiropractic and Osteopathic College of Australasia, *CAA* Chiropractors’ Association of Australia

^a^Australasian Academy of Functional Neurology; Chiropractic Australia; College of Chiropractic Neurodevelopment Paediatrics; Chiropractic Education Australia; American Chiropractic Neurology Board; Peninsula Chiropractic Union; Japanese Chiropractic Association

Over the past 12 months, participants had a variety of roles and may have had multiple roles (see Table 3).

**Table 3:** Roles of participants

Role	Number	Percent
University	42	34
Clinical supervision	36	29
Private Practice	106	85
Research	29	24
Volunteer work	35	28
Professional org. activities	43	35

(*N* = 125)

(N.B. Cumulative % > 100 as some participants nominated more than one role)

### Indigenous-specific item results

To meet the aims of this research, participants were asked about their experience working with Indigenous patients. Overall, 60% (*n* = 75) of participants reported they had previously treated Indigenous patients, leaving 24% (*n* = 30) reporting they had not treated Indigenous patients, while 9% (*n* = 11) were unsure. Only 3.4% (*n* = 4) asked patients if they identified as Aboriginal and/or Torres Strait Islanders and the remaining 96.6% (*n* = 114) did not ask their patients if they did or did not identify as Indigenous.

Over half of the participants working in a clinical setting (61%, *n* = 72), reported their level of comfort in working with Indigenous people as 10/10 on a Likert scale (where 1 corresponded to no comfort and 10 to completely comfortable) and 83% scored 8/10 and above (*n* = 97%). Of the practitioner respondents (*n* = 118), 17% (*n* = 21) had attended training to work with Indigenous patients, and 91% (*n* = 104) would be likely or very likely to attend training if made available.

Participants were asked should opportunities exist for them to provide chiropractic care in established Indigenous settings would they be interested in working there. Sixty-two percent (62.7%) (*n* = 74) replied they would, while 30% (*n* = 36) replied maybe, and 6.8% (*n* = 8) reported they would not be interested. Thirteen (10%) participants did not answer this question. Participants’ comments regarding their interest and readiness in working with Indigenous patients underwent a thematic analysis. Four themes emerged that shaped their interest in working with Indigenous patients: the chiropractor`s ‘personal situation’ such as travelling time to certain Indigenous organisations; their ‘reluctance to participate due to financial considerations including negotiating Medicare payments and remuneration; ‘circumvention’ such as wanting to become familiarised with cultural practices and having a “good clinical supervisor” in the case of students; and ‘positivity’ as expressed through respondents` appreciation for the “privilege of treating Indigenous people”. These themes, in turn, reinforced the perceived facilitators and barriers to the provision of a chiropractic service from the perspective of the chiropractor that the thematic analysis revealed as evidenced in Tables 4 and 5 below.

Participants were asked to consider barriers for Indigenous people seeking chiropractic care. There were 112 responses (90%) and these also underwent thematic analysis resulting in the identification of four broad thematic areas (see Table 4).

**Table 4:** Barriers to Indigenous patients seeking chiropractic care

Themes				
	**Personal** Financial	**Accessibility** Process Issues	**Educational** Lack of knowledge about chiropractic in Indigenous community	**Community Issues** Lived experience of institutionalised racism
**Examples of Comments**				
1	Cost of treatment	Location of Indigenous people/clinicians	Poor understanding in all communities re chiropractic	Reluctance of Indigenous patient/s to approach unknown clinician/clinic
2	Funding arrangements	Distance	Lack of awareness	Cultural rules of potential patients
3	Poor referring system	Reliable service in rural locations	No history of treatment in community/family	Fear of being treated in a manner that goes against cultural rules
4	Lack of proper equipment	Limited public transport		Fear of being treated badly
5	Time poor			

(*n* = 112)

(Note: Bold text refers to themes and text below refers to codes that were used to construct themes)

Participants were asked to consider the facilitators for Indigenous people seeking chiropractic care. There were 112 responses (90%) and these also underwent thematic analysis grouping into 4 themes (see Table 5).

**Table 5:** Facilitators to Indigenous patients seeking chiropractic care

Themes				
	**Personal** Financial	**Accessibility** Geographical Issues	**Educational** Education of Community	**Professional Issues** Cultural Competency
**Examples of Comments**				
1	Needs to be a reduction in cost of treatment to patients by increasing Extended Primary Care (EPC)/Medicare rebate	Provision of remote health services	Advertise benefits of chiropractic care	Train chiropractors in cultural competency and Indigenous health
2	Educating other health providers	Travelling chiropractors	Word of mouth for patients	Increase number of Indigenous chiropractors
3	Government to provide better subsidies for Indigenous patients	Community outreach programs	Educate community about synergy between chiropractic and Indigenous core beliefs	

(*n* = 112)

(Note: Bold text refers to themes and text below refers to codes that were used to construct themes)

## Discussion

This study investigated self-evaluation of a healthcare profession in cross-cultural preparedness, specifically by looking at the chiropractic profession and its self-perception of issues when working with Australian Indigenous peoples. It explored the extent to which chiropractors routinely collect data that identify Aboriginal and/or Torres Strait Islander patients, chiropractors’ clinical experience and participation in Indigenous healthcare and their interest in attending cultural competency programs to equip them with the necessary knowledge, skills and attitude to achieve best practice in chiropractic Indigenous healthcare. More than half of survey respondents thought that they had previously treated Indigenous (Aboriginal and/or Torres Strait Islander) people. The majority of respondents reported that they felt confident to treat Indigenous people. In this context it is interesting to note that chiropractors felt that one of the barriers and facilitators for Indigenous patients to access a chiropractor was geographical accessibility (see Tables 4 and 5 (location of Indigenous people/clinicians versus the provision of remote services, travelling chiropractors, community outreach programs)). Given that recent Australian Bureau of Statistics (ABS) [12] figures estimated that 35% of Aboriginal and Torres Strait Islander people lived in major cities, 45% lived in inner and outer regional areas and only 20% lived in remote and very remote areas this view of geographical location of Aboriginal and Torres Strait Islander people points to an added learning opportunity for chiropractors to recognise that Indigenous people may well live in their midst and that a respectful request whether an individual identifies as Indigenous becomes part of routine initial patient data collected.

The main points of dissonance revealed by this study included participants’ perceived high level of experience in treating Indigenous people and capturing identification of indigeneity at point of care. For example, 60% of respondents said that they had treated, or do treat Indigenous people, but only 4% asked their patients if they identify as Indigenous. It is unclear how the 56% ‘knew’ they had treated an Indigenous person, unless their indigeneity was known to the individual chiropractor at a personal level, as it is not possible to guess ethnicity by outward appearances [24].

The majority of participants (83%) scored 8–10 out of 10 in levels of ‘comfort’ or confidence in working with Indigenous people. While this seems to be a positive data point, it is important to consider the level of ‘comfort’ an Indigenous person might feel attending a chiropractor who claims to be completely at ease with an Indigenous person. Other studies demonstrate that to say ‘I treat all the people the same’ is never appropriate [25, 26] – a professionally competent chiropractor would not treat a patient presenting with acute back pain the same as a patient presenting with chronic hip pain. In the same vein, it is not culturally proficient to treat all patients as though they fit into the milieu of the mainstream majority. Highlighting the need for training in cultural competency is reflected in the finding that only 17% of respondents had undertaken any cultural competency training.

An important question that arises from this finding is whether the experience of members of Indigenous Communities reflects the chiropractors’ self-appraisal of a high level of comfort in treating (‘working with’) Indigenous people. A previously conducted study involving one of this papers' co-authors [27] reported that almost 50% of Indigenous people in a rural Community experiencing musculoskeletal pain had not accessed any treatment. The most common reasons or barriers given in this study were that ‘… they had learnt to live with the problem’, that ‘they were unaware of treatment options’ and there was also a belief that ‘private therapies were too expensive’ (see p 9–10). There is also an increasing awareness and acknowledgement by researchers who are interested in developing appropriate interventions for the management of chronic disease for Indigenous Communities that local knowledge and cultural issues are important determinants for acceptance of the intervention by Indigenous people [28]. What is meant by local knowledge and cultural issues is local lore, local cultural norms, understanding local context and respectfully engaging with the local Community. As an example it is well accepted that Australian Indigenous health issues are often divided into ‘men’s business’ and ‘women’s business’ and this therefore requires health professionals to take into account these cultural protocols [28]. Browne et al. also point to a “… complex interplay of social, political and economic determinants that influence …access to health care’ by Indigenous peoples [29]. What is evident from the literature is that Indigenous perspectives about barriers to access of health services are ‘poorly represented and undervalued …” [30]. It therefore is incumbent on chiropractors who desire to provide chiropractic care to members of Indigenous Communities to develop cultural competency in this area.

### Indigenous Australian cultural competence

Cultural competence, as a term, has been described and defined by Indigenous peoples. The definition of cultural competence recommended by Universities Australia [31] is:

*Cultural competence is the awareness, knowledge, understanding and sensitivity to other cultures combined with a proficiency to interact appropriately with people from those cultures in a way that is congruent with the behaviour and expectations that members of a distinctive culture recognise as appropriate among themselves. Cultural competence includes having an awareness of one's own culture in order to understand its cultural limitations as well as being open to cultural differences, cultural integrity and the ability to use cultural resources. It can be viewed as a non-linear and dynamic process which integrates and interlinks individuals with the organisation and its systems. (Indigenous Higher Education Advisory Council (IHEAC), 2007, p.5 and pp.34-38, and amended in 2011, IHEAC meeting and endorsed by the IHEAC Chair and Deputy Chair)* [32]*.*

### The importance of cultural competency

Despite the World Health Organisation’s ‘Closing the Gap in a Generation’ [7] program and the response of the Australian Government’s ‘Close the Gap’ programs [33], there are still serious discrepancies in health outcomes and life expectancies between Indigenous and non-Indigenous Australians [34]. Even though the social determinants of health contribute greatly to the burden of disease in Indigenous communities [35, 36], one of the most significant causes of continued poor health in Indigenous communities is direct racism [37], and indirect racism [38]. While health care providers may have little influence on turning the tide of racism in society, all practitioners need to be aware of their own level of skill in cultural competence.

Having a strong skill set in working with Indigenous people, including respectful communication and trust will enable the development of therapeutic relationships between practitioner and Indigenous patients [39]. For this reason, creating skilled health practitioners with cultural competence, which, in turn, leads to ‘proficiency’ to interact appropriately with Indigenous peoples [31] is seen as key to reducing inequalities in healthcare within Australia [40].

This study had several limitations. Although we invited participation in our project from all members of the CAA (Victoria) and used snowballing to include chiropractors of other associations and non-affiliated practitioners, our respondents may have only included those with an interest in this topic leading to a selection bias and limiting the generalisability of the results to other chiropractors. This, in turn, may have led to a bias in our results – particularly regarding chiropractors’ experience in treating Indigenous patients as well as their level of comfort in treating Indigenous people. These preliminary findings have nonetheless established a base upon which to refine and deliver a more representative study that surveys chiropractors nationally and provided the researchers with data supporting the potential benefit of developing and making available a cultural proficiency program for chiropractors.

Given the geographical location of chiropractors throughout Australia, it may have been instructive to record the postcode of participating chiropractors that completed the survey. Such a request would not preclude the anonymity associated with this study and would have provided the study team with an understanding of whether those practitioners having experience with treating Indigenous patients was associated with their geographical location.

Another limitation of this study is that it collected self-perception data from chiropractors. Though conclusions drawn from these data are necessarily limited, the study does provide preliminary information which may be used to inform on-going education of chiropractors and other health professionals. Future studies could compare whether the experience of members of the Indigenous Community match the chiropractors` self-appraisal. That is, do the chiropractors` perceptions of barriers and the thematic evaluation match the perceptions of the Indigenous Community? If not, what biases might be at play for chiropractors? If they do match, is this different to other health professions? Even if chiropractors are equipped with cultural competence, does the Indigenous Community want access to chiropractic services?

Although outside the scope of this article, future studies could explore whether chiropractors who were interested in working in Indigenous settings have different "interests" related to working with Indigenous patients compared to those who were not interested in this work, or were they the same?

Finally, a comparison of thematic evaluation between the results provided by chiropractors and an Indigenous response sampling is an important subsequent step. An analysis of these questions would create a worthwhile template by which other groups could challenge their preparedness to communicate with diverse cultural groups and their needs.

## Conclusions

The gap between Indigenous and non-Indigenous health equalities is a persistent problem in Australia. This inequality includes the enormous burden of illness presented by musculoskeletal conditions and perceived barriers to providing musculoskeletal healthcare to Indigenous Australians. A lack of formal cultural competency training of health professionals is thought to contribute to the lack of progress in closing the gap, including in musculoskeletal conditions.

Given the substantial burden of musculoskeletal illness experienced by Indigenous Australians, there may be an opportunity for chiropractors to become more culturally proficient in managing these conditions by participating in Indigenous cultural competency programs that enable them to engage respectfully, confidently and more capably with Aboriginal Australians. However, what has also become evident from this preliminary study are the potential questions that arise such as ‘How do chiropractors know if their patients are Indigenous or non-Indigenous if the majority of practitioners do not ask their patients to identify their indigeneity? Do they simply ‘guess’ their ethnicity based on outward appearances? Also why do chiropractors feel equipped to care for Indigenous patients when 83% have had no special training? Does the experience of members of the Indigenous community match ‘chiropractors’ self-appraisal of their ability to be culturally competent? Finally, to avoid potential bias, future studies should incorporate a larger, more representative sample that is generalisable. Incorporating measures that will address these questions may be used as steps to exploring cross-cultural pathways in caring for Indigenous Australians and aid in the development and implementation of cultural proficiency training programs for chiropractors.

## Additional file

Additional file 1: Survey Instrument. (DOCX 17 kb)

## Abbreviations

ABS: Australian bureau of statistics
CAA: Chiropractors’ association of Australia
CAAV: Chiropractors’ association of Australia (Victoria)
COCA: Chiropractic and osteopathic college of Australasia
IHEAC: Indigenous higher education advisory council
LBP: Low back PainRMIT, Royal Melbourne Institute of Technology

## References

[1] Rosenstock A, Mukandi B, Zwi AB, Hill PS (2013). Closing the Gaps: competing estimates of Indigenous Australian life expectancy in the scientific literature. Aust N Z J Public Health.

[2] Australian Institute of Health and Welfare (2014). Australia's health 2014. Chapter 7 Indigenous health [Internet].

[3] Lin IB, O'Sullivan PB, Coffin JA, Mak DB, Toussaint S, Straker LM (2012). 'I am absolutely shattered': the impact of chronic low back pain on Australian Aboriginal people. Eur J Pain.

[4] Vindigni D (2005). Promoting the musculoskeletal health of Indigenous Australians living in rural Communities. Aboriginal Health in Aboriginal Hands [PhD].

[5] Anderson I, Crengle S, Kamaka ML, Chen T-H, Palafox N, Jackson-Pulver L (2006). Indigenous health in Australia, New Zealand, and the Pacific. Lancet.

[6] Gracey M (2014). Why closing the Aboriginal health gap is so elusive. Intern Med J.

[7] Marmot M, Friel S, Bell R, Houweling TAJ, Taylor S (2008). Closing the gap in a generation: health equity through action on the social determinants of health. Lancet.

[8] Gibson O, Lisy K, Davy C, Aromataris E, Kite E, Lockwood C (2015). Enablers and barriers to the implementation of primary health care interventions for Indigenous people with chronic diseases: a systematic review. Implement Sci.

[9] Deeble J. Assessing the health service use of Aboriginal and Torres Strait Islander peoples [Internet]. Canberra: Commonwealth of Australia; 2009. Available from: http://citeseerx.ist.psu.edu/viewdoc/download?doi=10.1.1.560.7211&rep=rep1&type=pdf. Accessed 26 Apr 2017.

[10] Ware VA. Improving the accessibility of health services in urban and regional settings for Indigenous people [Internet]. Canberra: Australian Institute of Health and Welfare; 2013. Available from: http://www.aihw.gov.au/uploadedFiles/ClosingTheGap/Content/Publications/2013/ctgc-rs27.pdf. Accessed 26 Apr 2017.

[11] Australian Health Practitioner Regulation Agency. Chiropractic regulation at work in Australia, 2014/15: Chiropractic Board of Australia / AHPRA; 2016. Available from: http://www.chiropracticboard.gov.au/News/2016-04-08-chiropractic-regulation.aspx. Accessed 26 Apr 2017.

[12] Australian Bureau of Statistics (2014). Estimates and projections, Aboriginal and Torres Strait Islander Australians, 2001 to 2026 [Internet].

[13] Chapman R, Smith T, Martin C (2015). Qualitative exploration of the perceived barriers and enablers to Aboriginal and Torres Strait Islander people accessing healthcare through one Victorian Emergency Department. Contemp Nurse.

[14] Durey A, Thompson SC, Wood M (2012). Time to bring down the twin towers in poor Aboriginal hospital care: addressing institutional racism and misunderstandings in communication. Intern Med J.

[15] Baba JT, Brolan CE, Hill PS (2014). Aboriginal medical services cure more than illness: a qualitative study of how Indigenous services address the health impacts of discrimination in Brisbane communities. Int J Equity Health.

[16] Ruben BD (1989). The study of corss-cultural competence - traditions and contemporary issues. Int J Intercult Relat.

[17] Lie DA, Lee-Rey E, Gomez A, Bereknyei S, Braddock CH (2011). Does cultural competency training of health professionals improve patient outcomes? A systematic review and proposed algorithm for future research. J Gen Intern Med.

[18] Campinha-Bacote J (2002). The process of cultural competence in the delivery of healthcare services: A model of care. J Transcult Nurs.

[19] Betancourt JR, Green AR, Carrillo JE, Ananeh-Firempong O (2003). Defining cultural competence: a practical framework for addressing racial/ethnic disparities in health and health care. Public Health Rep.

[20] Vaismoradi M, Turunen H, Bondas T (2013). Content analysis and thematic analysis: Implications for conducting a qualitative descriptive study. Nurs Health Sci.

[21] Sadler GR, Lee H-C, Lim RS-H, Fullerton J (2010). Recruitment of hard to reach population subgroups via adaptations of the snowball sampling strategy. Nurs Health Sci.

[22] K C (2014). Constructing grounded theory.

[23] Fereday J, Muir-Cochrane E (2006). Demonstrating rigor using thematic analysis: A hybrid approach of inductive and deductive coding and theme development. Int J Qual Methods.

[24] Fforde C, Bamblett L, Lovett R, Gorringe S, Fogarty B (2013). Discourse, deficit and identity: Aboriginality, the race paradigm and the language of representation in contemporary Australia. MIA.

[25] Meiklejohn JA, Adams J, Valery PC, Walpole ET, Martin JH, Williams HM (2015). Indigenous cancer care in Queensland, Australia: Health professionals' framing of. Australian. J Cancer Nurs.

[26] Tang SY, Browne AJ (2008). ‘Race’matters: racialization and egalitarian discourses involving Aboriginal people in the Canadian health care context. Ethn Health.

[27] Vindigni D, Griffen D, Perkins J, Da Costa C, Parkinson L (2004). Prevalence of musculoskeletal conditions, associated pain and disability and the barriers to managing these conditions in a rural, Australian Aboriginal community. Rural Remote Health.

[28] Barnett L, Kendall E (2011). Culturally appropriate methods for enhancing the participation of Aboriginal Australians in health-promoting programs. Health Promot J Austr.

[29] Browne AJ, Smye VL, Rodney P, Tang SY, Mussell B, O'Neil J. Access to primary care from the perspective of Aboriginal patients at an urban emergency department. Qual Health Res. 2010;21(3):333–48. 10.1177/1049732310385824 21075979

[30] Peiris D, Brown A, Cass A (2008). Addressing inequities in access to quality health care for indigenous people. Can Med Assoc J.

[31] Universities Australia, Indigenous Higher Education Advisory Council (2011). National Best Practice Framework for Indigenous Cultural Competency in Australian Universities [Internet].

[32] Indigenous Higher Education Advisory Council (2008). Ngapartji-Ngapartji - yerra : stronger futures : report of the 3rd annual IHEAC Conference 21 November 2007 Adelaide.

[33] Cooper D (2011). Closing the gap in cultural understanding: social determinants of health in Indigenous policy in Australia.

[34] Australian Indigenous HealthInfoNet. Overview of Australian Aboriginal and Torres Strait Islander health status 2015. 2016. Available from: http://www.healthinfonet.ecu.edu.au/health-facts/overviews. Accessed 26 Apr 2017.

[35] Carson B, Dunbar T, Chenhall RD, Bailie R (2007). Social determinants of Indigenous health.

[36] Osborne K, Baum F, Brown L. What Works?: A Review of Actions Addressing the Social and Economic Determinants of Indigenous Health. Closing The Gap Clearing House [Internet]. 2013 [cited 2016 June 9]. Available from: http://www.aihw.gov.au/uploadedFiles/ClosingTheGap/Content/Publications/2013/ctgc-ip07.pdf. Accessed 26 Apr 2017.

[37] Larson A, Gillies M, Howard PJ, Coffin J (2007). It's enough to make you sick: the impact of racism on the health of Aboriginal Australians. Aust N Z J Public Health.

[38] Paradies Y, Harris R, Anderson I (2008). The impact of racism on Indigenous health in Australia and Aotearoa: Towards a research agenda.

[39] Durey A, Thompson SC (2012). Reducing the health disparities of Indigenous Australians: time to change focus. BMC Health Serv Res.

[40] Bainbridge R, McCalman J, Clifford A, Tsey K. Cultural competency in the delivery of health services for Indigenous people. Closing the Gap Clearinghouse [Internet]. 2015 [cited 2016 May 22]. Available from: http://apo.org.au/node/56408. Accessed 26 Apr 2017.

