# Supplementary material for: Chiropractors` experience and readiness to work in Indigenous Australian Communities: a preliminary cross-sectional survey to explore preparedness, perceived barriers and facilitators for chiropractors practising cross-culturally
Source: Chiropr Man Therap. 2017 May 2;25:13. doi: 10.1186/s12998-017-0144-0 (PMC5414224; doi:10.1186/s12998-017-0144-0)
Supplement: Additional file 1: — Survey Instrument. (DOCX 17 kb) [file 12998_2017_144_MOESM1_ESM.docx]

Appendix 1 Survey Instrument

Q1 What is your age in years?

______ Your age: (1)

Q2 What is your gender?

- Male (1)
- Female (2)

Q3 Are you currently in private chiropractic practice?

- Yes (1)
- No (2)
- If yes, how many years? (3) ____________________

Q5 What is your highest level of qualifications?

- Diploma (1)
- Advanced Diploma (2)
- Bachelor (or Double Bachelor) (3)
- Masters (4)
- PhD (5)

Q6 Are you a member of any of the following professional chiropractic organisations?

- CAA (1)
- COCA (2)
- None (3)
- Others (please specify) (4) ____________________

Q8 Indicate all of the roles in which you have been involved as a chiropractor over the last 12 months

- University (1)
- Research (2)
- Clinical supervision (3)
- Volunteer work (4)
- Private practice (5)
- Professional organisation activities (6)

Q9 Do you treat or have you previously treated Indigenous patients?

- Yes (1)
- No (2)
- Unsure (3)
- Comment (4) ____________________

Q10 Do you ask patients if they identify as Aboriginal and/or Torres Strait Islander on your clinic  personal details sheet?

- Yes (1)
- No (2)

Q11 How comfortable are you in working with Indigenous patients? (with 1 being not at all comfortable, and 10 is completely comfortable)

- 1 (1)
- 2 (2)
- 3 (3)
- 4 (4)
- 5 (5)
- 6 (6)
- 7 (7)
- 8 (8)
- 9 (9)
- 10 (10)

Q12 If opportunities exist/ed for you to provide chiropractic care in an established Indigenous setting such as an Aboriginal Health Service, would you be open to participating ? Comments?

- Yes (1) ____________________
- No (2) ____________________
- Maybe (3) ____________________

Q13 Have you ever attended any training in working with Indigenous patients?

- Yes (1)
- No (2)

Q14 What barriers, if any do you perceive for Indigenous people accessing chiropractic treatment?

Q15 In your opinion, what factors would enhance Indigenous people accessing chiropractic care?

Q16 How likely would you be to attend training if it was offered as part of your continuing professional development ?

- 1 (1)
- 2 (2)
- 3 (3)

Q17 Any additional comments?
